# Digital nerve injuries: a review of predictors of sensory recovery after microsurgical digital nerve repair

**DOI:** 10.1007/s11552-012-9433-1

**Published:** 2012-06-28

**Authors:** Joline F. Mermans, Bas B. G. M. Franssen, Jan Serroyen, Rene R. W. J. Van der Hulst

**Affiliations:** 1 Department of Plastic, Reconstructive and Hand Surgery, Maastricht University Medical Centre, Peter Debyelaan 25, 6229 HX Maastricht, The Netherlands; 2 Department of Methodology and Statistics, Research School CAPHRI, Maastricht University, Peter Debyeplein 1, 6200 MD Maastricht, The Netherlands

**Keywords:** Conduits, Digital nerve, Grafts, Neurorrhaphy, Sensory recovery

## Abstract

**Background:**

Optimal surgical management of digital nerve lesions remains uncertain despite the publication of numerous studies. The purposes of this review were primarily to analyze whether there is a superior surgical technique for digital nerve repair and secondarily to statistically verify the variables to be predictors of sensory recovery.

**Methods:**

A literature search was performed using PubMed including citation from MEDLINE. Studies were included if they involved patients with digital nerve lacerations in whom end-to-end neurorrhaphy, nerve grafts, conduits, or end-to-side neurorrhaphy were performed. Further, the sensory outcome had to be assessed according to the modified American Society for Surgery of the Hand guidelines to stratify for two-point discrimination in millimeters. The variables age, follow-up, delay in repair, type of trauma, and gap length were extracted. The association between each predictor and response was assessed using a linear mixed model and corrected for heterogeneity between studies. Significance was considered present at *p* ≤ 0.05.

**Results:**

Of the 34 articles found, 14 articles were included giving appropriate individual data for 191 nerves. There was no statistically significant difference in outcome between operation techniques. Age and follow-up were verified predictors of sensory recovery.

**Conclusion:**

In this review, the type of operation for digital nerve repair does not influence sensory outcome. However, we verified outcome to be influenced by the patient’s age and the follow-up period. To add more scientific evidence to our results, larger cohort prospective studies need to be done with better detailed description of data.

## Introduction

Digital nerve lacerations are common in hand trauma surgery and are even the most frequently injured of the peripheral nerves. Digital nerve injury can result from simple cuts or from severe hand trauma. In case a digital nerve is left unrepaired, the axonal growth will disperse and could lead to neuroma formation which then would interfere with rehabilitation, functional recovery, and sensory deficit especially in the thumb and the second digit which are involved in pinch and gnostic function [43]. Sensibility is an essential factor, concerning a normal hand function. Since Moberg [36] studied and described the sensory function of the fingers, many studies reported the treatment and the evaluation of the sensibility after treatment of nerve injuries.

Primary repair by end-to-end neurorrhaphy can be performed in about 82 % of the cases [28]. Brown recommended primary repair when there is a clean, sharply incised wound and both nerve ends are easily seen and mobilized without extension of the wound and with availability of a well-trained team with adequate facilities [10]. In about 18 % of the cases, when gaps exist, nerve reconstruction is required by grafting or tubulization [28]. The gold standard for these nerve type injuries is the nerve autograft [9, 27, 32, 38, 46]. Usually, the sural nerve or the antebrachial cutaneous nerve serve as favorable donor nerves to bridge the nerve defect. However, the morbidity caused by sacrificing a functioning nerve resulted in searching for suitable non-autologous graft materials. Effective alternatives were found, like synthetic and autogenous non-nervous conduits which function as a guide for axonal sprouting, a barrier against scar tissue ingrowths and maintain an internal milieu for nerve regeneration [26]. Recently, the end-to-side neurorrhaphy has been described to solve avulsion injuries or when the proximal stump is not available for traditional end-to-end repair. In 1991, this type of neurorrhaphy was reintroduced by Viterbo et al. and reconnects a distal injured nerve stump laterally to a neighboring nerve [2, 7, 17, 28, 51, 52].

Predictors of sensory recovery have been evaluated in several studies. Weinzweig et al. [57] found mechanism of injury and age to be predictors of sensory recovery after digital nerve injury [1, 3, 16, 20, 31, 41, 47, 57]. They did not observe a significant correlation between gender, involved digit, level of injury, time from injury till repair, and gap length. Numerous other studies did state that there is a correlation between the latter factors and sensory recovery. However, none of these studies performed a statistical analysis to confirm an actual correlation between these factors.

In conclusion, we noticed a lack of consensus regarding the most optimal management of digital nerve repair. Also, it is remarkable that there is still no agreement on which variables are predictors of a successful sensory recovery. The aim of this review is to present an update and statistical evidence for digital nerve repair. We review the surgical techniques, the outcomes, and the variables to be predictors of sensory recovery.

## Materials and Methods

A literature search was performed in March 2011 using the PubMed service that includes citations from MEDLINE and other life science journals. Initial search focused on the text words: digital nerve, trauma, injury, surgery, repair, and sensory. In a secondary search, the medical subject heading term “peripheral nerve” was also used. The limit was set on abstracts in English, Dutch, German, French, and Spanish dating back to 1980. If the title of a study was relevant, the abstract was reviewed by the first and second author. Full-text articles were obtained and reviewed if the abstracts suggested that the study met the inclusion criteria. In addition, footnote chasing of references cited in previous studies of sensibility outcome in patients with digital nerve repair was performed. The studies were classified according to their level of evidence [12].

Articles were eligible for inclusion if they met the inclusion criteria. Inclusion criteria included (1) patients with clinically identified peripheral digital nerve lesion, (2) the surgical intervention was either microsurgical end-to-end neurorrhaphy, end-to-side/terminolateral neurorrhaphy, non-nervous conduits repair, or grafts surgical repair, and (3) the outcome assessment was postoperative sensory recovery in static two-point discrimination (s2PD) expressed in millimeters and stratified into groups according to the modified American Society for Surgery of the Hand guidelines (modified ASSH) (Table 1) [4]. Studies were excluded when they (1) did not meet the eligibility criteria, (2) did not present the individual data per patient, or (3) were descriptive reviews.

**Table 1:** Modified ASSH guidelines for stratification of s2PD results

Rating	s2PD (mm)
Excellent	<6
Good	6–10
Fair	11–15
Poor	>15 or protective sensation

Following the literature analysis, it was possible to reanalyze the individual data per patient. We extracted the next variables from each study meeting the inclusion criteria: the type of repair, the age at the time of injury, the type of injury, the delay between injury and repair in weeks, the gap length in centimeters, the follow-up period in months, and the sensory outcome expressed in millimeters. None of these articles presented individual data for all mentioned variables. Therefore, we included articles with minimal six out of seven variables. We did not include the variables gender and site of injury because, for this, data was not specified per patient most of the time.

In addition, we performed a statistical analysis of individual data from the selected articles. First, the association between each predictor and response was assessed separately using a linear mixed model. The above-described risk factors were all analyzed on *p* value, 95 % confidence interval, estimate, and standard error of the mean. Further, the variables that were significantly associated with sensory recovery (*p* ≤ 0.05) were included in a linear mixed model to evaluate their independent contribution to the prediction of recovery. The linear mixed model was chosen because we needed to correct for correlation between subjects from the same study. The heterogeneity between different studies could affect the relation between risk factors and outcome.

## Results

### Trial Selection

**Fig. 1:**
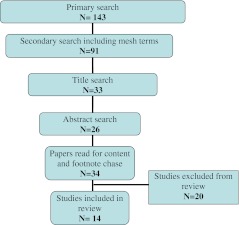
Flow diagram of the selection process of included articles

A total of 34 papers were recovered after the PubMed search. Among these, 20 did not meet the inclusion criteria because of different reasons (Table 2). Eventually, 14 studies were appropriate for inclusion. A total of 191 nerves were evaluated for sensory outcome. The numbers of nerve repair for each operation technique were 21 with grafts, 71 with end-to-end neurorrhaphy, 33 with biological conduits, 42 with synthetic conduits, and 24 with end-to-side/terminolateral neurorrhaphy. The selection process is described in Fig. 1. The 20 excluded studies are mentioned in Table 2 with the description of each article and the reason of exclusion in Tables 3, 4, 5, 6, and 7.

**Table 2:** Excluded articles

Study	Year
Altissimi et al. [3]	1991
Berger et al. [8]	1991
Efstathopoulos et al. [16]	1995
Wang et al. [55]	1996
Weinzweig et al. [57]	2000
Cheng et al. [13]	2001
Tadjalli et al. [48]	1995
Al-Ghazal et al. [1]	1994
Kallio et al. [24]	1993
Goldie et al. [19]	1992
Mackinnon et al. [30]	1988
Weber et al. [56]	2000
Battiston et al. [6]	2006
Rinker et al. [42]	2011
Walton et al. [54]	1989
Tang et al. [50]	1993
Risitano et al. [43]	2002
Meek et al. [33]	2004
Meek et al. [34]	2004
Mennen [35]	2003

**Table 3:** End-to-end primary repair group studies excluded

Study	Year	Follow-up method	Predictors	Not predictors	Type of study
Altissimi et al. [3]	1991	No individual data	Age	Postoperative treatment	Prospective
			Time to repair (primary versus delayed)		
			Type of trauma (sharp versus crush)		
Berger et al. [8]	1991	No individual data	Age	Associated injuries	Prospective
			Surgeon experience		
Efstathopoulos et al. [16]	1995	No individual data	Age	Presence of associated injuries	Retrospective
			Type of injury (sharp versus crush)		
Wang et al. [55]	1996	No individual data	Primary versus secondary repair	Contralateral nerve block	Prospective
			Age		
			Injury mechanism		
Weinzweig et al. [57]	2000	No individual data	Age (child versus adult)	Gender	Retrospective
			Type of injury (sharp versus avulsion	Involved digit	
				Level of injury	
				Radial or ulnar side	
				Time injury til repair (acute versus delayed	
Cheng et al. [13]	2001	No individual data	Tactile stimulation after repair	Mechanism of injury	Prospective randomized
			Age	Site of injury	
Tadjalli et al. [48]	1995	No individual data, British scale	Severity of injury	–	Retrospective
Al-Ghazal et al. [1]	1994	Highet method and no individual data	Smoking	Associated injuries	Retrospective
			Type of injury (sharp versus crush)		
Kallio et al. [24]	1993	No individual data	Age	Level of injury	Retrospective
			Time of repair (acute versus delayed)	Type of trauma	
Goldie et al. [19]	1992	No individual data	–	No correlation between the used assessments tests	Retrospective

**Table 4:** PGA conduit group studies excluded

Study	Year	Follow-up methods	Predictors	Not predictors	Type of study
Mackinnon et al. [30]	1988	Lack of individual data	−	Type of tube	Prospective
				Contralateral nerve block	
Weber et al. [56]	2000	No individual data	Type of repair (PGA versus nerve grafts and primary repair)	Age	RCT
			Gap length	Location of injury	
			Mechanism of injury	Worker’s compensation status	
				Time to repair	
Battiston et al. [6]	2006	m2PD	Type of repair (autogenous versus synthetic conduits)	Type of tube	Prospective
				Age	
Rinker et al. [42]	2011	No individual data	Type of repair (PGA versus vein conduits)	Type of tube	RCT
			Smoking		

*m2PD* moving two point discrimination in millimeters

**Table 5:** Autogenous conduit group studies excluded

Study	Year	Follow-up methods	Predictors	Not predictors	Type of study
Walton et al. [54]	1989	m2PD	Time till repair (acute versus delayed)	Laceration versus avulsion	Retrospective
Tang et al. [50]	1993	m2PD	Secondary repair with vein conduits after primary repair	–	Prospective
Battiston et al. [6]	2006	m2PD	Type of repair (autogenous versus synthetic conduits)	Type of tube	Prospective
				Age	
Risitano et al. [43]	2002	Mackinnon and Dellon no millimeters given	Vein grafts satisfactory for gap length <30 mm, poor for gaps >30 mm	–	Retrospective

*m2PD* moving two point discrimination in millimeters

**Table 6:** Nerve graft group studies excluded

Study	Year	Follow-up methods	Predictors	Not predictors	Type of study
Meek et al. [33, 34]	2004	No individual data/review	PGA permits reconstruction of longer defects	–	Literature review of peripheral nerve repair
			Incorporation of muscle in vein superior results than vein conduits alone in same defect		
Wang et al. [55]	1996	Not enough individual data	Primary versus secondary repair	Length of graft	Prospective
			Age	Contralateral nerve block	
			Injury mechanism		
Berger et al. [8]	1991	No individual data	Age	−	Prospective
			Surgeon experience		
			Tension on nerve graft		
			Associated injuries		
Kallio et al. [24]	1993	No individual data	Age	Level of injury	Retrospective
			Time of repair (acute versus delayed)	Type of trauma	
			Length of nerve graft

**Table 7:** End-to-side group study excluded

Study	Year	Follow-up methods	Predictors	Not predictors	Type of study
Mennen [35]	2003	Highet British Medical Research Council	Elimination of tension	–	Prospective
			Postoperative immobilization		

### Assessment of Level of Evidence

The 14 included studies had a design classification of II to IV. Five studies were level II, two studies were level III, and seven studies were level IV.

### Description of Included Studies

Table 8 provides the clinical characteristics of the 14 included studies, which incorporates the amount of repaired nerves, the study design, the study year, and the type of repair. All studies were published between 1985 and 2010. The patient cohort age ranged from 8 to 72 years, with a mean of 39.52 years. The patient cohort follow-up time after surgery ranged between 3 and 144 months, with a mean of 28.31 months. The patient cohort time till repair ranged between 0 and 148 weeks (mean, 3.28 months). The nerve cohort gap length ranged between 0 and 4 cm, with a mean of 1.16 cm. An excellent sensory outcome was achieved in 25.13 % of the patients. Table 9 gives a crude association between the predictor age and an excellent sensory recovery.

**Table 8:** Included studies

Author	Year	No. of nerves repaired	Age (years)		Follow-up (months)		Delay (weeks)		Type of repair	Type of study
			Mean	Range	Mean	Range	Mean	Range		
Hirasawa et al. [21]	1985	12	29.4	8–47	33.1	19–70	1.1	0–8	End-to-end/graft	Prospective
Segalman et al. [44]	2001	19	65	60–72	>12	–	–	–	End-to-end	Retrospective
Sullivan et al. [47]	1985	42	–	20–65	13	9–96	5.86	0–88	End-to-end	Retrospective
Marcoccio et al. [32]	2010	21	38	11–61	43	18–96	Delayed (not further specified)		Autogenous conduit	Retrospective
Lee et al. [26]	2008	3	32.33	19–52	103	33–144	48.8	2.42–104	Autogenous conduit	Case series
Norris et al. [37]	1988	8	40.9	15–61	7.63	3–11	68	0–120	Grafts	Prospective
Nunley et al. [39]	1990	19	27.77	16–52	55.15	24–89	22.15	0–44	Grafts	Prospective
Bushnell et al. [11]	2008	11	33	18–55	11	12–22	–	–	Synthetic conduit	Prospective
Lochmeyer et al. [28]	2009	12	38	12–66	12	–	21.6	0–148	Synthetic conduit	Prospective
Battiston et al. [7]	2007	19	40.05	15–67	29.7	6–74	5.5	0–72	Synthetic conduits	Prospective
Artiaco et al. [5]	2010	7	45	20–62	35. 71	8–60	19.42	0–48	End-to-side	Prospective
Pelissier et al. [40]	2001	6	32.83	13–46	12.2	6–15	–	–	End-to-side	Retrospective
Frey et al. [18]	2003	2	–	12–42	–	37–48	–	–	End-to-side	Case series
Voche et al. [53]	2004	10	30	9–55	16.8	9–29	0	0–0	End-to-side	Retrospective

**Table 9:** Crude association of predictors

Predictors	Groups	Excellent sensory recovery, % (*n*)^a^
Age (years)	<16	36.4 (4/11)
	16–25	21.7 (5/23)
	26–40	25.5 (12/47)
	>40	29.4 (20/68)
	Total no.	149
Gap length (cm)	<1	32.2 (27/84)
	>1	21.8 (19/87)
	Total no.	26.9 (46/171)
Follow-up (months)	<6	0 (0/7)
	6–12	26.9 (17/63)
	>12	25.6 (31/121)
	Total no.	191
Trauma	Sharp	27.8 (27/97)
	Avulsion/circular saw	28.3 (17/60)
	Iatrogenic	0 (0/5)
	Secondary	0 (0/2)
	Total no.	164
Delay	No delay	23.6 (13/55)
	1 day–1 month	26.6 (8/30)
	1–6 months	12 (3/25)
	6–12 months	30 (4/12)
	>12 months	27.3 (3/11)
	Total no.	133
Type of surgery	End-to-end	31 (22/71)
	Biological conduits	24.3 (8/33)
	Synthetic conduits	31 (13/42)
	End-to-side	8.3 (2/24)
	Grafts	14.3 (3/21)
	Total no.	191

^a^Results of this table are not adjusted for the type of studies included in this review

### Risk Factors Associated with Sensory Recovery

For sensory recovery, the type of surgery did not significantly influence outcome (*p* = 0.707). A longer time of follow-up after trauma was predictive for better recovery. The estimated average monthly decrease in 2PD is 0.035 (slope = −0.035). This reduction in 2PD is statistically significant (*p* = 0.042) (Table 10).

**Table 10:** Result adjusted for type of surgery

Parameter	Estimated change in s2PD	*p* value	95 % CI
Follow-up	−0.034961	0.042	[−0.068565–(−0.001357)]
Age	0.057806	0.040	[0.002590–0.113022]
Gap length	1.067788	0.120	[−0.280003–2.415578]

A younger age is significantly predictive for better sensory recovery. For every year, the estimated average increase of 2PD is 0.058 (slope = 0.058). This increase in 2PD is statistically significant (*p* = 0.040) (Table 10).

A broader gap length, although borderline significant (*p* = 0.120), showed a tendency for a worse sensory recovery. For every centimeter of gap length, the estimated average increase in 2PD is 1.07 (slope = 1.07) for all types of operations (Table 10).

In the second analysis, we tested age adjusted for follow-up and type of surgery. The 2PD increases over time (slope = 0.066). This increase in 2PD is highly statistically significant (*p* = 0.017) (Table 11). There was no significant difference in mean sensible outcome in regard to time to repair (*p* = 0.803) and type of trauma (*p* = 0.165).

**Table 11:** Results age adjusted for follow‐up and type of surgery

Parameter	Estimated change in s2PD	*p* value	95 % CI
Age	0.066475	0.017	[0.012612–0.120337]

## Discussion

The aim of this review was to provide an update of the current surgical interventions for digital nerve repair. With this, we wanted to analyze whether there is evidence for the superiority of one technique above the other and statistically verify the prognostic factors for sensory recovery after reconstruction.

Our study showed no significant difference in sensory recovery between the types of operation for digital nerve repair. There are only two randomized controlled trials (RCTs) comparing surgical interventions for digital nerve repair. One of these, recently published by Rinker et al. studied 76 nerves randomized in two groups. One group underwent repair with a vein conduit and the other group underwent repair with polyglycolic acid conduits (PGA) [42]. The other RCT, published by Weber et al., studied 136 digital nerve transections. They randomized the patients into two groups, the first consisting of end-to-end neurorrhaphy or nerve grafts and the second consisting of PGA conduits repair [56]. Rinker et al. observed no difference in sensory recovery between both groups [42]. In contrast and not in line with our paper, Weber et al. did find a significant difference between groups. They found improved sensory outcome when using a conduit for nerve gaps of 4 mm or less, compared with end-to-end repair. They further showed a significantly better sensory recovery for synthetic conduits compared to nerve-grafted repairs with a gap length of 8 mm or more [56]. The reason Weber et al. [56] did observe a difference, unlike us, may be explained due to the type of studies included in this review, which were all level of evidence II to IV. Battiston et al. presented their personal clinical experience on tubulization repair of digital nerves, using biological and synthetic tubes. Good clinical results were seen in both groups [6].

In the past decade, numerous studies proposed several variables to predict sensory recovery [1, 16, 47, 57]. We observed a tendency of worse sensory recovery with broader gap length. This seems logical because a bigger distance for axonal growth has to be bridged, even though the type of surgery is the important predictive factor in case of gap length. Literature states that if a gap has to be bridged, tensionless repair with end-to-end neurorrhaphy can be difficult and, as a result, other types of surgical interventions have to be performed [11, 22, 28, 32]. The critical distance for nerve regeneration through a conduit was reported as 1.0 to 1.5 cm in a rat model [49]. In our review, 65 % of the nerve gaps were below 1.5 cm. The critical distance for nerve regeneration in humans has not yet been established. According to Walton et al. [54], Chiu et al. [14], and Mackinnon et al. [30], the results of digital nerve reconstruction with nerve grafts were comparable to those of nerve defects repaired by a vein conduit for defects <3.0 cm [14, 30, 54]. These results and the small sample size for nerve gaps above 3 cm in our data (4 %) could explain why we found no significant difference in sensible recovery between vein conduits and synthetic conduits in gap length above and under 1 cm.

A trend towards better outcome for sharp injuries was observed. The nonsignificance of this result could be explained according to the variable gap length. The type of trauma is related to the gap length that has to be bridged; as in big crush injuries, normally there is more tissue damage and, as a consequence, after trimming the dead nerve ends, a bigger gap length is left for repair. The reason why the variable gap length was not significant has been discussed above.

No association between delay in repair and outcome was seen. Weinzweig et al. [57] and Weber et al. [56] did neither find any correlation between time from injury to repair and outcome. On the other hand, they did find a significant correlation between type of trauma and outcome at a young age. In the older patient, this correlation disappeared [48, 56, 57].

In general, age was found to be a main factor for sensory recovery [1, 3, 8, 16, 25, 41, 47]. Berger et al. [8] and Steinberg et al. [45] emphasize that the better results in young patients are not only caused by a better axonal regeneration and a greater adaptability [8, 44, 45]. They suggest that older patients have fewer receptors because of age correlated centrally occurring changes, as uninjured digits in younger patients compared with older patients have superior sensibility [8, 31, 44, 56].

Finally, follow-up is verified to be positively correlated with sensory recovery. The recovery period of an injured nerve is based on functional reorganizational changes in the somatosensory cortex, mainly because of misdirection of regenerating axons. Regardless of how accurate the repair technique is, axonal misdirection is unavoidable [23, 29, 58]. According to the literature, a stable level of sensibility is reached by 6 months, which could be the time needed for functional reorganization. According to our crude analysis, excellent results are only seen after a period of 6 months [5, 13, 31].

Our review study differs from most others cited in the literature because we incorporated a statistical analysis from the included data. Most reviews in the literature are descriptive reviews and none of them covers all of the above-described available operation techniques for digital nerve injuries [5, 33, 57].

In addition, we want to mention that we included the end-to-side/terminolateral group because we wanted to give a comprehensive overview of all operation techniques used for digital nerve repair in the past 30 years. While documentation concerning the end-to-side/terminolateral group experience is limited, we still found 24 repairs performed according to this technique. Therefore, we think it is important to include these results in our analysis. We realize though that it is not a first-choice technique when doing a digital nerve repair. But Artiaco et al. [5] reported their clinical experience along with a literature review of digital nerve repair with the end-to-side technique, and the technique has encouraging results. It can be used in case of loss of substance and as an alternative to biological and synthetic conduits when digital nerve repair by means of nerve autograft is declined by the patient.

In conclusion, we state that the type of surgery, if selected accurately, does not influence the sensory outcome in our analysis but we verified that the time of follow-up and age of injury are predictive factors concerning the sensibility. However, this study still has a few limitations. Firstly, one major limitation is the reduced number of included nerves in this study. To perform a statistical analysis, and with this to obtain a good functional comparison of sensibility after digital nerve reconstruction, a uniform outcome measurement has to be used. Most authors survey the ASSH to stratify the results of s2PD but other surveys rely on the criteria set by the Nerve Injuries Committee of the British Medical Research Council, as modified by Mackinnon and Dellon to stratify the results for s2PD and moving two-point discrimination (m2PD) [28]. For this study, we intentionally choose the s2PD to be our uniform measurement outcome. The s2PD is a valid and reliable measurement of functional sensibility [15]. Despite the fact that most surveys used this measurement as an expression of their outcome, we had to exclude a significant amount of nerves from studies using Highet and Sander’s criteria as modified by Mackinnon and Dellon if they did not specify the exact value of s2PD and m2PD in millimeters.

Secondly, most studies performed concerning this subject were of small sample size, lacking statistical analysis, or with a low level of evidence (level III or IV). Therefore, it was not possible to only include studies of a higher sample size or higher level of evidence as this would reduce our sample size to a minimal level. Finally, we excluded the articles without presentation of individual data which could have led to selection bias if other predictors of recovery were present in the excluded patients.

Therefore, as a final and major remark, we want to point out one important recommendation for future research in this area. We think that a large cohort of patients should be followed up and randomized in the different types of operation techniques with a better detailed description of individual data in order to find answers to the lacunas in knowledge regarding digital nerve repair.
